# Computational Mutagenesis and Inhibition of *Staphylococcus aureus* AgrA LytTR Domain Using Phenazine Scaffolds: Insight From a Biophysical Study

**DOI:** 10.1155/2024/8843954

**Published:** 2024-09-18

**Authors:** Prince Manu, Prisca Baah Nketia, Priscilla Osei-Poku, Alexander Kwarteng

**Affiliations:** ^1^ Department of Chemistry Kwame Nkrumah University of Science and Technology, Kumasi, Ghana; ^2^ Kumasi Centre for Collaborative Research in Tropical Medicine Kwame Nkrumah University of Science and Technology, Kumasi, Ghana; ^3^ Department of Biochemistry and Biotechnology Kwame Nkrumah University of Science and Technology, Kumasi, Ghana

**Keywords:** AgrA LytTR domain, antibiotic resistance, binding energies, computational mutagenesis, molecular dynamics simulations, phenazine scaffolds, *Staphylococcus aureus*, virulence

## Abstract

Biofilm formation by *Staphylococcus aureus* is a major challenge in clinical settings due to its role in persistent infections. The AgrA protein, a key regulator in biofilm development, is a promising target for therapeutic intervention. This study investigates the antibiofilm potential of halogenated phenazine compounds by targeting AgrA and explores their molecular interactions to provide insights for drug development. We employed molecular docking, molecular dynamics simulations, and computational mutagenesis to evaluate the binding of halogenated phenazine compounds (C1 to C7, HP, and HP-14) to AgrA. Binding free energy analysis was performed to assess the affinity of these compounds for the AgrA-DNA complex. Additionally, the impact of these compounds on AgrA's structural conformation and salt bridge interactions was examined. The binding-free energy analysis revealed that all compounds enhance binding affinity compared to the Apo form of AgrA, which has a *Δ*G_bind_ of −80.75 kcal/mol. The strongest binding affinities were observed with compounds C7 (−113.84 kcal/mol), HP-14 (−115.23 kcal/mol), and HP (−112.28 kcal/mol), highlighting their effectiveness. Molecular dynamics simulations demonstrated that these compounds bind at the hydrophobic cleft of AgrA, disrupting essential salt bridge interactions between His174-Glu163 and His174-Glu226. This disruption led to structural conformational changes and reduced DNA binding affinity, aligning with experimental findings on biofilm inhibition. The halogenated phenazine compounds effectively inhibit biofilm formation by targeting AgrA, disrupting its DNA-binding function. The study supports the potential of these compounds as antibiofilm agents and provides a foundation for rational drug design targeting the AgrA-DNA interaction. Future research should focus on further optimizing these lead compounds and exploring additional active sites on AgrA to develop novel treatments for biofilm-associated infections.

## 1. Introduction

Persistent infections often originate from bacterial biofilms, which are characterized by their pathogenic properties. According to data from the National Institutes of Health (NIH), biofilm formation is implicated in 65%–80% of microbial and chronic diseases [1–3]. Ali et al. [4] indicate that bacterial biofilms typically resist the human immune system and drugs, significantly limiting the efficacy of existing antimicrobial agents [5–7]. Understanding biofilm formation in bacteria is crucial for controlling and eradicating diseases associated with bacterial biofilms.

*Staphylococcus aureus* (*S. aureus*) is a ubiquitous bacterium that is a leading cause of a wide range of infections in both community and healthcare settings. While often present as a harmless colonizer on the skin and mucous membranes, *S. aureus* can rapidly transition into a formidable pathogen, causing conditions ranging from mild skin infections to life-threatening diseases such as pneumonia, bloodstream infections, and endocarditis [8].

The impact of *S. aureus* infections on public health cannot be overstated, as they contribute significantly to morbidity and mortality worldwide. Over the years, the mortality rates attributed to *S. aureus* infections have been a cause for concern among healthcare professionals and policymakers. Reports from the United States demonstrate that *S. aureus* infections result in seven million hospitalizations, indicating significant harm caused by hospital-acquired methicillin-resistant *S. aureus* (HA-MRSA) [9]. *Staphylococcus* commonly forms biofilms on medical implants and devices, leading to device malfunction and increased mortality rates [10]. Despite efforts to improve treatment protocols and infection control measures, the mortality associated with *S. aureus* infections remains unacceptably high, especially in vulnerable populations and those with compromised immune systems. Understanding the factors contributing to the lethality of these infections is crucial for devising effective strategies to mitigate their impact on public health. In *S. aureus*, most virulence factors are regulated by accessory gene regulator (Agr)–mediated quorum sensing.

The response regulator AgrA is pivotal in biofilm formation and virulence production since it controls the expression of virulence factors by binding to promoter sites. The Agr operon in *S. aureus* consists of Promoters P2 and P3, which encode transcripts RNA II and RNA III, respectively [11]. RNA II facilitates the expression of AgrD (precursor of autoinducing peptide), AgrB (involved in AIP maturation and export), AgrC (which phosphorylates AgrA upon AIP reaching a threshold), and AgrA (which binds to P2 and P3 promoters to initiate Agr operon restart) [12]. AgrA-driven expression of RNA III promotes the expression of major virulence factors and hemolytic exotoxins from hla and hld genes [13]. Additionally, the expression of various hemolysins, enterotoxins, lipases, leukocidins, and phenol-soluble modulins (PSMs) is regulated by AgrA binding to the P3 promoter and RNA III expression [11, 14] and bacteria oxidation stress regulation [15]. AgrA can also directly bind to promoters and initiate PSM expression, which possesses cytolytic activity independent of RNA III [16]. Another virulence factor, coagulase production, is also under Agr's control, with coagulases contributing to the pathogenesis of *S. aureus* infections such as osteomyelitis [17]. The quorum-sensing system Agr is known to regulate biofilm detachment in *S. aureus*; however, the molecular detachment components of *S. aureus* that are regulated by quorum sensing are yet unknown. With this, mutation and deletion studies on the AgrA protein have been investigated [18–22]. Results from these studies highlight the importance of AgrA in biofilm regulation and pathogenicity as a result of *S. aureus* infection. Mutations of amino acids responsible for DNA binding and total deletion of the AgrA protein result in halting biofilm formation and accompanied pathogenesis in animal models. Hence, with this important role played by AgrA, it is important to find small-molecule inhibitors that can inhibit the biological function of the protein. Since few studies have been conducted in this field, this study also seeks to contribute to the existing literature.

In order to solve the persistent pathogenesis caused by *S. aureus,* new interventions are urgently needed to help curb the problem. In light of that, the pathogen *Pseudomonas aeruginosa* has been reported to have the capacity to produce approximately 90%–95% of the blue phenazine pigment, pyocyanin, which also serves as a defense mechanism against other bacteria, preventing disruption of biofilms. Recognizing pyocyanin's potent antibacterial properties, Huigens, Abouelhassan, and Yang investigated microbial competition strategies and explored the potential of phenazine antibiotic-inspired compounds in eliminating persistent bacterial biofilms in *S. aureus* [23]. Their research led to the identification of a series of halogenated phenazines (HPs) that effectively eradicate bacterial biofilms in *S. aureus*, with future endeavors aiming to translate these initial findings into groundbreaking clinical applications for treating persistent biofilm infections. The impressive activities of the HP compounds towards the *S. aureus* and eradication of biofilm suggest that they may interact with AgrA as part of their inhibitory activities against the bacteria. In this study, the ability of these compounds to inhibit the action of AgrA was investigated using molecular docking, molecular dynamics (MD) simulation, and computational mutagenesis studies.

## 2. Methodology

### 2.1. Protein Selection and Preparation

The 3D configuration of the *S. aureus* AgrA LytTR domain protein, bound to DNA and a Mg^2+^ ion, was acquired from the Protein Data Bank (https://www.rcsb.org) under the PDB ID: 3BS1 [24]. Upon retrieval, water molecules and heteroatoms were removed from the protein structure. Histidine residues were protonated, and partial charges were introduced to achieve charge balance within the protein structure. The backbone structures underwent energy minimization using AMBERff4SB, and Gasteiger charges were computed using an antechamber in chimera [25, 26]. This approach was to attain a prepared protein complex with the DNA for molecular docking.

### 2.2. Ligand Preparation

Ligand preparation was employed to subject the modeled compounds to structure optimization, geometry equilibration, and compute quantitative structure activity calculations. Ligand modeling was performed using Spartan'14 (Wavefunction Inc., Irvine, California, United States), and structural optimizations and energy minimization were performed using the density functional theory B3LYP/6-31G∗ basis set [27]. The coordinates of the compounds were saved in .sdf and .pdb formats.

### 2.3. Evolutionary Profile Analysis

Evolutionary profiling aids in pinpointing important residues responsible for the biological functions of macromolecules. The evolutionary significance of amino acids, crucial for the protein's biological function and structure, was assessed using the webserver (http://consurf.tau.ac.il). The evaluation involved determining the amino acids' importance based on an evolutionary profile. The parameters chosen for phylogenetic analysis included the CSI-BLAST homologous search algorithm, with three iterations and an *E*-value cut-off of 0.0001. The protein database UNIREF-90 was utilized, with 150 reference sequences selected and a maximum sequence identity of 95%. Additionally, a minimum identity for counterparts of 35% was set. The Bayesian alignment method and MAFFT-L-INS-i calculation method were employed, along with the selection of the best evolutionary substitution model [28].

### 2.4. Molecular Docking

The molecular docking approach in computational studies is relevant since it provides the initial findings of ligand affinities and ascertained binding sites.

#### 2.4.1. Virtual Screening

The virtual screening was performed with PyRx v0.824 [28, 29]. We employed the AutoDock Vina28 Lamarckian Genetic algorithm and empirical free energy scoring function within the PyRx v0.8 interface to minimize the protein. The .sdf format of the ligands considered for this study was also subjected to energy minimization considering these parameters: forcefield, mmff94; optimization algorithm, steepest descent; and a considered total number of steps, 200,000. The energy-minimized ligands were then subjected to screening against the selected protein in a blind docking manner. A total of nine different poses were generated for the ligands against the protein, and the binding affinities of the lowest energy poses were recorded.

#### 2.4.2. Computational Mutagenesis of Residues Responsible for DNA Binding

Mutagenesis studies are important since they highlight the specific importance and biological roles played by amino acid residues. In this study, mutations were done on amino acid residues responsible for DNA binding.

The UCSF Chimera software (https://www.cgl.ucsf.edu) was employed for the mutagenesis of the relevant residues. These residues, Arg233, Asn201, and His169, necessary for DNA binding were considered and mutated to alanine. The structures obtained via mutations were subjected to MD simulations for further studies.

### 2.5. MD Simulation Study and Post-MD Analysis

MD simulations were employed to evaluate the probability of maintaining binding poses obtained from molecular docking studies and to investigate newly discovered intermolecular interactions. The initial structures utilized were derived from the molecular docking investigations.

MD simulations lasting 200 ns were conducted on all compounds bound to the AgrA protein, including the DNA-bound, mutated proteins, and apoprotein states. These simulations were carried out using GROMACS v.2018.6 [30], executed on the Lengau cluster at the Center for High-Performance Computing in Cape Town, South Africa. Ligand topologies were generated via the CGenFF (https://cgenff.umaryland.edu/) web server, while protein topology was derived from the CHARMM36 all-atom force field [30]. Solvation was achieved using the TIP3P water model within a cubic boundary box, with sodium and chloride ions added to maintain system neutrality. Energy minimization was performed to alleviate steric clashes via the steepest descent algorithm. Subsequently, systems underwent NVT and NPT equilibration under constant number, volume, pressure, and temperature ensembles, reaching temperatures up to 300K and a pressure of up to 1 bar over 100 ps. Long-range electrostatic interactions were computed using the particle mesh Ewald (PME) method, with Coulomb and van der Waals interaction cut-offs set to 1.2 nm. The production run utilized a time step of 2 fs, with coordinate trajectories saved every 10 ps. Three-dimensional periodic boundary conditions (PBC) were applied throughout the simulation, ensuring that the systems remained within the defined cubic boundary box.

Following the MD simulations, trajectory analyses of the complexes were conducted using the simulation output files to assess their stability. Key parameters including root mean square deviation (RMSD), root mean square fluctuation (RMSF), and radius of gyration (RoG) were computed and graphed using XMGRACE to evaluate stability. Salt bridge analysis was performed with the visual molecular dynamics (VMD) plug-in engine tool, employing a 3.2 Å oxygen–nitrogen distance cut-off. Principal component analysis (PCA) of both the complexes and the apoprotein was carried out based on the RMSD of the protein backbone and the RMSF of the C_*α*_ atoms. Using the resulting PCA data of the proteins, the free energy landscape (FEL) was calculated based on their PC1 and PC2 eigenvalues and eigenvectors, respectively. Furthermore, trajectory video analysis was conducted using VMD 1.9.345, utilizing both the structural (.gro) and PBC compressed trajectory (.xtc) files for all complexes obtained from the MD simulations.

### 2.6. Clustering of Protein Structures

The simulation generates numerous structural frames, which are then grouped into clusters based on their structural differences from a reference structure. Backbone atoms of the protein structures are utilized for superposition and clustering purposes since this helps to attain the most average structural conformation of the protein in the bound and unbound states.

The GROMOS algorithm [28] was employed, along with a clustering RMSD cut-off of 0.10 nm. The clustering algorithm identifies structures within this RMSD cut-off and forms clusters accordingly. The process involves selecting the structure with the highest number of neighbors within the RMSD cut-off, treating it as a cluster, and removing it from consideration. This procedure is repeated for the remaining structures. From each trajectory, the central structure of the highest-ranked cluster is chosen as the cluster representative for further analysis.

### 2.7. Molecular Mechanics Poisson–Boltzmann Surface Area (MM-PBSA) Binding Energy Calculations

The study utilized the MM-PBSA method [31] to assess the energetic stability of ligands bound to receptors. This approach analyzed various energetic components, such as bond angles, torsional energies, van der Waals and electrostatic interactions, and solvation effects, both polar and nonpolar, using an implicit solvation model. The calculations also considered configurational entropy related to complex formation. Overall binding energies were estimated by combining molecular mechanical potential energy (*E*_MM_) with parameters for polar (*G*_pol_) and apolar (*G*_apol_) solvation. The binding energy (*E*_binding_) of the system is estimated as follows:

(1) $$\begin{array}{c} E_{\text{binding}}=E_{\text{complex}}-E_{\text{AgrA}+\text{DNA}}+E_{\text{ligand}} \end{array}$$

(2) $$\begin{array}{c} E_{\text{binding}}=E_{\text{complex}}-E_{\text{AgrA}}+E_{\text{DNA}} \end{array}$$

where *E*_complex_ is the total free energy of the AgrA-DNA ligand complex and *E*_AgrA−DNA_ and *E*_ligand_ are the total free energies of the individual target (receptor) and ligand in a solvent, respectively. The individual binding free energy of each component is expressed as follows:

(3) $$\begin{array}{c} E_{\text{binding}}=E_{\text{MM}}+G_{\text{solv}} \end{array}$$

where *E*_mm_ represents the molecular mechanics energy terms and *G*_solv_ represents the solvation energy terms. The entropic factor (ΔTS) was omitted from the calculation due to its significant computational complexity. Furthermore, studies indicate that the overall impact of the entropic component is often negligible [30]. This decision underscores why the term “binding energy” (*E*_binding_) is used instead of Δ*G* in this context. The *E*_mm_ is made up of all bonded and nonbonded energies in the system and, thus, can be expressed as follows:

(4) $$\begin{array}{c} E_{\text{MM}}=E_{\text{bonded}}+E_{\text{nonbonded}}=E_{\text{bonded}}+E_{\text{vdW}}+E_{\text{elec}} \end{array}$$

where *E*_bonded_ is the bonded interactions consisting of bond, angle, dihedral, and improper interactions. *E*_nonbonded_ represents the nonbonded interactions that include both electrostatic (*E*_elec_) and van der Waals (*E*_vdW_) interactions, which are calculated using the Coulomb and Lennard–Jones potential functions, respectively. The solvation energy term (*G*_solv_) is expressed as follows:

(5) $$\begin{array}{c} G_{\text{solv}}=G_{\text{polar}}+G_{\text{nonpolar}} \end{array}$$

where *G*_polar_ represents polar solvation energies and *G*_nonpolar_ is the nonpolar solvation energies. *G*_polar_, which is the electrostatic contribution, is calculated by solving the Poisson–Boltzmann equation. The nonelectrostatic term of solvation energy, *G*_nonpolar_, includes repulsive and attractive forces between solute and solvent generated by cavity formation and van der Waals interactions, respectively.

## 3. Results

### 3.1. Evolutionary Profile Analysis

**Figure 1:**
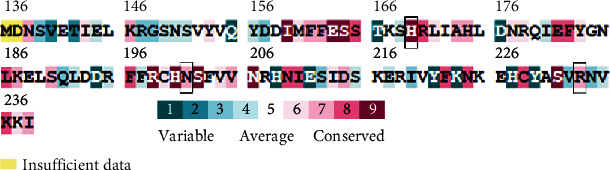
Evolutionary profile of AgrA LytTR protein with important residues needed for DNA binding, shown in black rectangles.

Results from the amino acid evolutionary profiling highlight the importance of some residues and how these residues are conserved among other proteins of the same family. Observations made from this study demonstrate that residues such as His169, Asn201, and Arg233 shown in the black box in Figure 1 are significantly conserved; hence, this means these residues are also important for protein function.

### 3.2. Screening of HP Compounds

**Figure 2:**
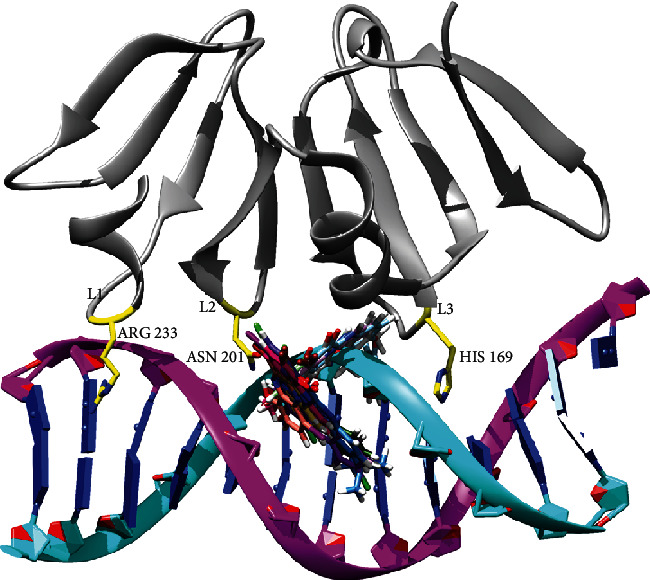
Binding site of selected ligands after high-throughput screening. Ligands bind at the hydrophobic interface needed for DNA binding.

We screened reported compounds from a study conducted by Huigens, Abouelhassan, and Yang [23], screened against the AgrA protein using AutoDock Vina in PyRx v0.8. Results from the high-throughput virtual screening also predicted the binding site of the ligands used for the study (Figure 2). The high-throughput virtual screening of the ligand library resulted in a broad range of binding affinities towards the DNA-binding interface of the AgrA LytTR domain. Some compounds, however, did not bind at the interface from this study using both tools. Both high-throughput tools predicted binding energies of the ligands ranging from −8.0 to −10.0 kcal/mol for the compounds. The binding energies are summarized in Table 1.

### 3.3. MD Simulation

MD simulations were performed to explore the stability of the protein−ligand complexes. MD simulations were performed for 200 ns on the compounds C1-C8, HP, and HP-14 to understand their binding mode. The overall stability was analyzed by RMSD, RMSFs, RoG, PCA, and FEL.

### 3.4. Ligand Stability and Interactions

**Figure 3:**
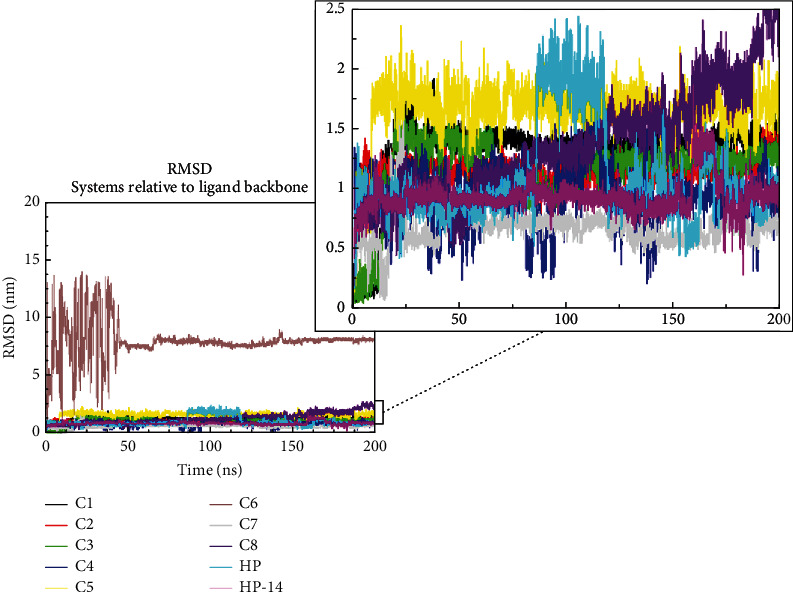
RMSD of ligands after 200 ns simulation. Showing the stability of the ligands considered for this study.

To assess the stability of the ligands when bound to the protein and to explore the favorable interactions necessary for binding, the RMSD of the ligands was evaluated (Figure 3).

The RMSD of the ligand backbone to the initial docking pose determines the stability of the ligand. From the results, all compounds were stable throughout the simulation period aside from C6. Compound C6 had a higher deviation at time 2 ns. This higher deviation was simultaneous through to 48 ns (Figure 3) between 5 and 13 nm. However, after 50 ns, C6 remained stable around 8.5 nm through to time 200 ns (Figure 3). Upon critical analysis of the compounds proposed as stable, it was realized that C8 was stable from 0 to 180 ns. After 180 ns, C8 revealed a slightly higher deviation (Figure 3 [zoom]) from 182 to 200 ns. Compound HP also had a deviation at time 95–125 ns with an RMSD of 2.0 nm (Figure 3). However, this observed deviation speculated HP as an unstable compound. Compounds C1 and C3 showed a deviation that is similar at 15 ns with RMSD values of 1.5 nm and 1.8 nm, respectively (Figure 3). Also, Compounds C2 and C4 had stable trajectories, as there were no deviations observed throughout the simulation period with RMSD values of 1.2 and 1.1 nm, respectively (Figure 3). Compound HP-14 also showed a stable trajectory until time 155–160 ns (Figure 3), where a slight deviation was observed. Among all the compounds considered for this study, comparing the ligand RMSD values, Compound C7 was the most stable with RMSD values of 0.75 nm (Figure 3).

**Figure 4:**
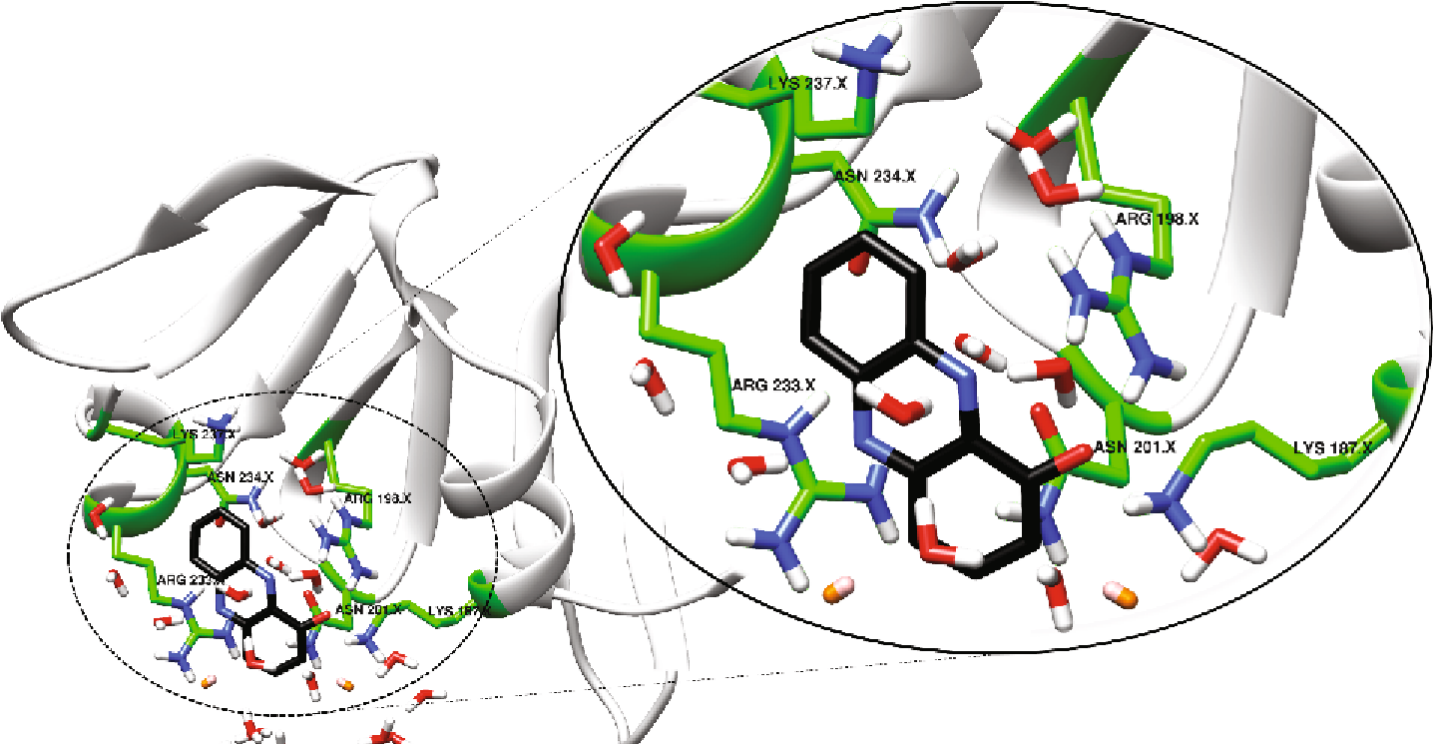
Binding site of HP ligand and interacting residues during the molecular dynamics simulations. HP interacted with residues responsible for DNA binding.

From Figure 4, it is evident that the major scaffold, HP, established interactions with amino acids such as Arg198, Arg233, Asn201, Asn234, Lys187, and Lys237.

**Figure 5:**
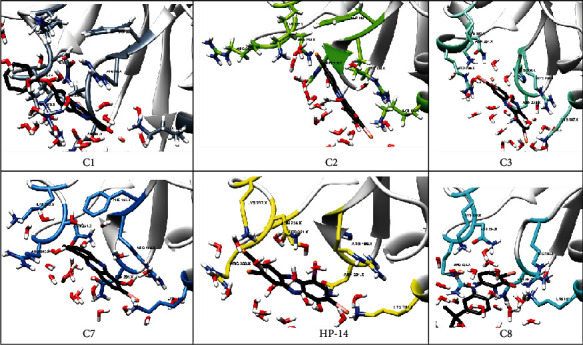
Binding site of ligands and interacting residues during the molecular dynamics simulations. Interactions between DNA binding residues and ligands were established.

Probing into the interactions established by the other compounds, it was observed that compounds C1, C2, C3, C4, C7, C8, and HP-14 mimicked the interactions observed for HP throughout the simulation (Figure 5 and Figure S1). However, some other amino acids interacted with the compounds, and these amino acids include His200, Phe197, and Ser231. Compound C5 binds to a different site from the binding site of the other compounds as well as the major scaffold (Figure S2). The hydrophobic and hydrogen bond interactions played significant roles in the binding of these compounds into the binding site of the AgrA protein.

### 3.5. Protein Stability and Conformational Changes

To assess the structural stability of the protein−ligand complex and to compare the conformational changes of the protein in its apo state and the presence of the ligand, the RMSD of the protein was calculated (Figure S3).

The RMSD of the backbone atoms to the initial docking structure determines the stability of the protein. The RMSD of the protein in the presence of C1–C8 was stable around 0.2–0.3 nm throughout the simulation time of 200 ns. The RMSD of the protein HP complex fluctuated up to 15 ns, and then it was quite stable around 0.4 nm. The RMSD of the protein HP-14 complex was stable up to 185 ns and then had a slight fluctuation through to the end of the simulation with an RMSD of 0.3 nm. In the case of the apo state, the protein was very stable throughout the simulation period with an RMSD value of 0.35 nm.

**Figure 6:**
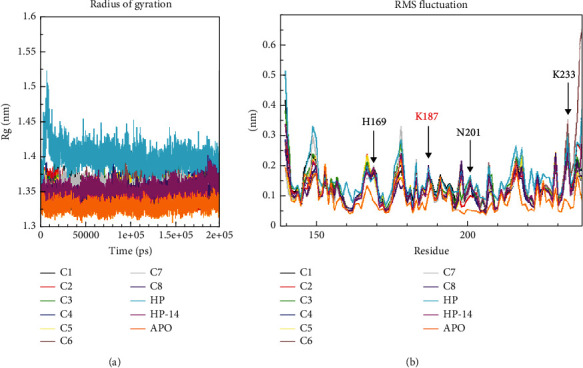
(a) Radius of gyration for proteins and (b) RMS fluctuations of amino acid residues. Rg and RMSF give a general overview of the protein structure.

To understand the conformational changes of the protein in the bound and unbound states, we investigated the Rg and RMSF of the systems used in this study. The profile for the compactness of the protein in the bound and unbound states is shown in Figure 6(a). From this analysis, the apo state was more compact as compared to the bound proteins. Especially, the protein HP complex shows a higher flexibility of the protein from 0 to 15 ns, and aside from this, it was evident that the protein had higher flexibility as compared to the apo state. Also, the profile of residual fluctuations upon ligand binding shows differences in residue behavior during the interactions with the ligands and even at the point where some ligands lost interactions with these residues. The analysis was done throughout the simulation, as shown in Figure 6(b). Higher fluctuations were observed for Asn201, His169, Lys187, and Lys233 for all unbound proteins as compared to the apoprotein.

**Figure 7:**
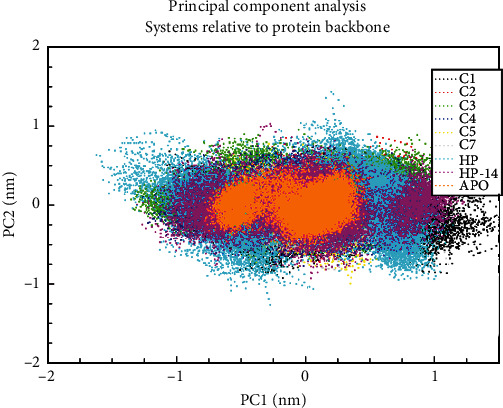
Principal component analysis of protein backbone considering the motions of the C_*α*_. Motions of the side chains were revealed considering the C_*α*_.

To understand the impact of ligand binding on the protein at the atomistic level, PCA was performed. The PCA considers the collective motion of atoms of the protein in the bound and unbound states. The MD trajectory was applied to a phase space to obtain an array of eigenvalues, which helped to clarify the protein's flexibility and stability [32]. A further analysis of the overall flexibility and stability was conducted using a diagonalized covariance matrix trace. More trace values indicate more model flexibility with the instability of the protein's average structure, and this result is consistent with our earlier RMSF and RMSD of the protein investigated. By conformationally sampling proteins and their complexes in phase space using a projection of the C-alpha atom, a scatter plot representing the tertiary conformations along Eigenvectors 1 and 2 was produced (Figure 7).

The results indicated that the collective motion of atoms in the apo state was not rampant as compared to the bound protein complexes. It is therefore evident that the ligands caused and increased the collective motions in the protein throughout the simulation. However, the compound HP caused higher collective motions when bound to the protein, as the other compounds also mimicked similar motions.

**Figure 8:**
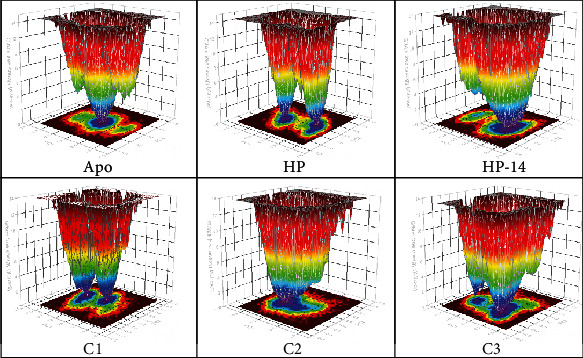
FEL evaluation of complexes from this study. Minima energy evaluation of the protein structure when ligands were bound to the protein. The violet color shows higher energy while red shows lower energy.

Using the first two principal components (PC1 and PC2) as coordinates, the FEL analysis of the protein was evaluated. An inbuilt GROMACS scripts (*g_covar*, *g_anaeig*, and *g_sham*) were used in evaluating the FEL of the protein. The FEL analysis was performed to look at the unique protein conformation in the bound and unbound states throughout the simulation. Examining the FEL plot indicates that higher energy states are indicated by red to yellow regions, while dark blue to violet labels reflect the energy minima and energetically favored structural conformation [33]. The data clearly show that the free energy in the global free energy minimum was changed by ligand binding. Furthermore, distinct global energy minima were noted in every structure, indicating stable and unstable states (Figure 8 and Figure S4).

**Figure 9:**
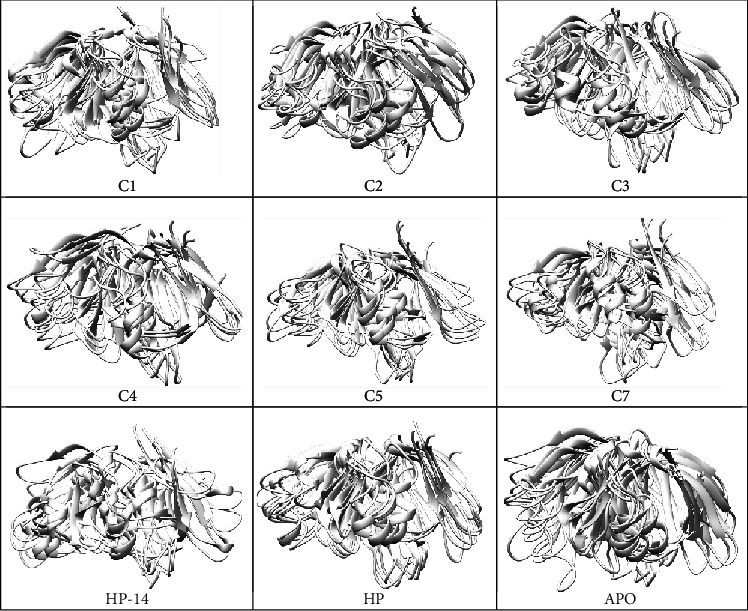
Clustering of similar structures throughout for structural conformational analysis.

Considering the FEL of the apoprotein, it was observed that the protein attained a stable energetically favored structural conformation throughout the simulation. In the case of the HP compound, there were two distinct energy minima of the protein conformation as the ligand was bound to it. A similar observation can be seen when C1 and C3 were bound to the protein. Compounds C2, C4, C5, C6, C7, and C8 showed slight distinct energy minima conformation of the protein when these compounds were bound to it.  Figure 9 shows the clustered conformations of the protein throughout the simulation period, as these conformations suggest the FEL evaluated for each system. Following the clustering of protein conformations, it was observed that loop regions of the protein had major fluctuations throughout the simulation. Comparing the apo state to the bound protein systems, it was clearly seen that the loop regions of the apo state had restricted motions at some point since the regions have residues responsible for DNA binding. The motions of these residues were not restricted in the bound systems.

### 3.6. Computational Mutagenesis of Residues Responsible for DNA Binding

**Figure 10:**
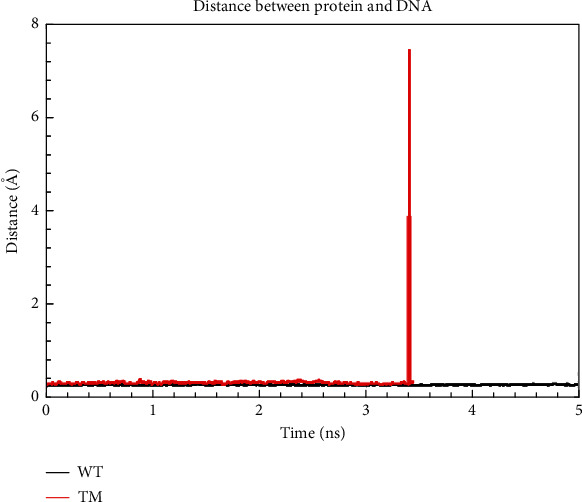
Distance between AgrA LytTR and DNA.

Our results support experimental data that highlights the importance of the residues that make direct contact with the DNA, particularly the roles played by His169, Asn201, and Lys233 for biofilm formation. Therefore, to evaluate this finding, we mutated the three residues to alanine and labeled it as triple mutant (TM). From the results obtained by determining the distance between the protein and the DNA, it was evident that the TM protein lost affinity for the DNA at time ~3.2 ns, while that for the WT was maintained (Figure 10). A similar scenario was observed for the bound protein complexes, where in this case, loss of affinity by the protein for the DNA was seen at 0.5 ns (Figure S5).

### 3.7. Salt Bridge Interaction Analysis

**Figure 11:**
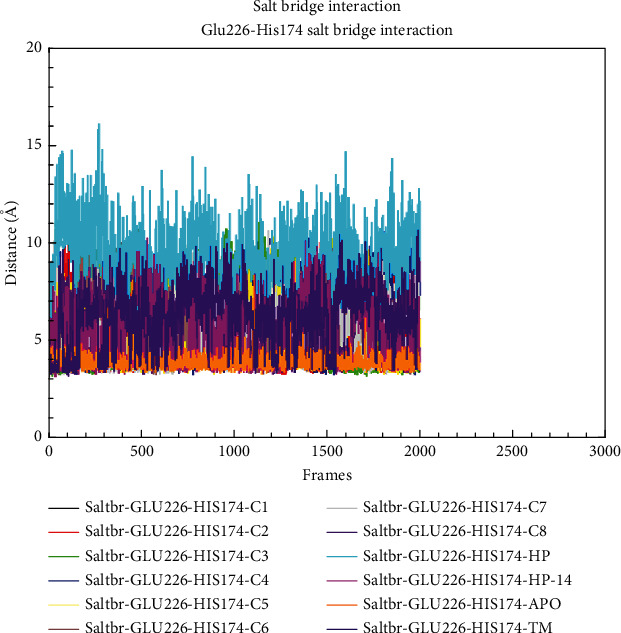
Distance between Glu2266-His174 salt bridge interactions throughout the simulation.

The salt bridge interactions are key components for protein folding. Residue His174 was shown to be involved in two salt bridge interactions with residues Glu163 and Glu226 in the crystal structure of the AgrA LytTR domain [24, 34]. The distance between His174 and the other residues involved in the salt bridge interactions was evaluated in the bound (wild type), unbound, and TM complexes. From the analysis, observations made were that variation in distance between the Glu163-His174 salt bridge interaction in the bound complexes was simultaneous between 4.9 and 6.5 Å, while that in both the wild type (apo) and the TM was significantly constant throughout the simulation at a distance of 4 Å (Figure S6). For Glu226-His174 salt bridge analysis, the wild-type system demonstrated a stable salt bridge interaction with a distance of 4.9 Å. In the bound and TM complexes, the case was different since a higher deviation in distance was seen throughout the simulation. The distance in the bound and wild-type complexes ranges from 5.5 to 14 Å, with the AgrA-HP complex demonstrating higher fluctuations (Figure 11).

### 3.8. MM-PBSA Binding Energy Calculations

The apo form (AgrA-DNA without any compound) has a moderate van der Waals interaction and electrostatic energy with a relatively high polar solvation energy, resulting in a binding free energy of −80.75 kcal/mol. C1 significantly enhances van der Waals interactions and decreases electrostatic contributions compared to the apo form, resulting in a binding free energy of −104.36 kcal/mol. C2 shows similar trends as C1, with slightly less favorable van der Waals interactions but a similar binding free energy of −103.77 kcal/mol. C3 has the highest van der Waals interaction among the first three compounds, leading to a binding free energy of −106.66 kcal/mol. C4 further improves the van der Waals interactions and polar solvation energy, resulting in a binding free energy of −107.36 kcal/mol. C5 maintains strong van der Waals and electrostatic interactions, leading to a binding free energy of −105.76 kcal/mol. C6 has the highest van der Waals interaction among the first six compounds, with a binding free energy of −110.58 kcal/mol. C7 exhibits the strongest van der Waals and electrostatic interactions, leading to a binding free energy of −113.84 kcal/mol, the most favorable among all the compounds tested. HP shows strong interactions similar to C7, with a binding free energy of −112.28 kcal/mol. HP-14 has the second most favorable binding free energy of −115.23 kcal/mol, with balanced contributions from all energy terms (Table 2).

Overall, the binding free energies of the AgrA-DNA complexes with the various compounds indicate that all the compounds (C1–C7, HP, and HP-14) enhance the binding affinity compared to the apo form. The binding affinities follow the trend: C7 > HP − 14 > HP > C6 > C4 > C3 > C5 > C1 > C2 > apo. The strongest binding is observed with C7, followed closely by HP-14 and HP. These results highlight the significant role of van der Waals and electrostatic interactions in stabilizing the complexes and provide insights into the design of potential inhibitors targeting the AgrA-DNA interaction.

## 4. Discussion

Resistance to antibiotics has always been a problem with *S. aureus*. The resistance of the pathogen to the existing drugs is a result of biofilm formation, and in the process, AgrA plays a key role in initiating transcription at both the P2 and P3 promoters upon activation.

AgrA operates as a dimer, with its dimerization being dependent on activation. Studies show that the binding of AgrA with acetyl phosphate leads to the conversion of AgrA monomers into dimers [35]. After the formation of a dimer, there is activation of the receptor's C-terminal LytTR DNA-binding domain (137-238) [36]. Previous findings from the crystallography investigation highlight the significance of the three loops (referred to as L1, L2, and L3), which penetrate two consecutive major grooves and the minor groove that divides them. Among these loops, three residues directly interact with DNA, and two of these residues are indispensable for facilitating the protein's DNA binding capability. Also in our study, we noted the importance of another residue from the *α*-helix that contributes to DNA binding (Figure S7 [in yellow color]), which is Lys187. Structural conformational analysis highlights that Lys187 is also involved in DNA binding. Interaction with Lys187 would cause inhibition by enabling protein loss affinity for DNA; therefore, findings from both the molecular docking and MD simulations indicate that the compounds established interaction with Lys187.

This defense mechanism enables the bacteria to be resistant to antibiotics. Inhibition of AgrA activity is therefore a promising strategy to disrupt biofilm formation and ultimately lead to its death since AgrA is involved in other biological activities [18–22] aside from biofilm formation. Therefore, there is a pressing need for the exploration and design of novel inhibitors with enhanced properties to effectively target and inhibit AgrA both in vitro and in vivo.

In the quest to identify other sources of biofilm quenching compounds, research demonstrates that *P. aeruginosa* secretes high concentrations of the phenazine antibiotic pyocyanin, a deep blue pigment, which is reported to be the primary eradicating agent of *S. aureus* and other bacteria [37, 38]. Currently, research on the antibacterial property of pyocyanin is exploring its activity against *S. aureus* and other bacteria, but little or no research is focused on its antibiofilm property. Huigens, Abouelhassan, and Yang however explored the antibiofilm activity of some HPs against *S. aureus* in an experimental study. [23] Whereas much is known experimentally, the molecular insights of the explored antibiofilm potency of these HPs are not known.

In this study, reported HP compounds with biofilm eradication properties were screened against the *S. aureus* AgrA protein. The virtual screening approach identified the interface of AgrA which is important for DNA binding, as the binding site of HP compounds (Figure 2). The results indicate that the compounds can hinder the function of the Agr system by blocking AgrA binding to DNA. From the docking results, the compounds established interactions with amino acid residues responsible for DNA binding in the presence and absence of the DNA chains. Some interactions were consistent with all the compounds, hydrogen bond interaction between the compounds and amino acid residues such as Arg233, Asn201, His169, and Lys187. With this, the compounds were proposed as potential inhibitors of AgrA binding to the DNA, knowing the importance of these residues.

From MD simulation analysis (Figure 2 and Figure S1), it is evident that the major scaffold, HP, and its other scaffolds such as C1, C2, C3, C4, C7, C8, and HP-14 established interactions with amino acids such as Arg198, Arg233, Asn201, Asn234, His200, Lys187, Lys237, Phe197, and Ser231. Other compounds like C5 made interactions with Arg170, Asn185, His169, and Tyr183. Amino acids such as Arg233, Asn201, and His169 have direct contact with the DNA, while other amino acids make indirect contact with the DNA. Mutagenesis studies revealed that point mutation of Arg233 and His169 to alanine, the protein is no longer able to interact with DNA. Another point mutation of Asn201 to alanine demonstrated that the affinity of the protein for the DNA is drastically reduced. Therefore, Asn201 is thought to stabilize the interaction between AgrA and the DNA backbone [11, 14, 24, 33, 36]. With the important roles played by these residues, interacting with these amino acids leads to a reduction of the protein's affinity for the DNA, hence leading to inhibition of biofilm formation.

Other researchers identified that the C-terminal loop of AgrA contains a hydrophobic cleft where fragment compounds (small molecules) can bind, leading to inhibition of the DNA-binding ability of the protein. This region contains residues Ser231, Val232, Arg233, Asn234, Lys236, Lys237, and Ile238. This loop is important for the DNA-binding activity of the protein as residue Arg233 is essential for the DNA-binding activity of the protein and residues Ser231, Val232, and Asn234 make indirect contact with the DNA. In addition, mutations in that region result in a partially deficient or complete lack of activity of the protein for DNA binding; hence, it is important to note that the compounds binding to this region will result in a loss of affinity for DNA binding. To support these findings, the region was identified as a putative DNA-binding region because of its hydrophobic nature in the first report of the LytTR domain [24, 34].

A newly discovered small-molecule inhibitor, named savirin, has been found to selectively target AgrA in *S. aureus*. This inhibitor effectively blocks the activation of the P3 promoter and the synthesis of RNA III by AgrA. Additionally, it hinders the DNA-binding function of the AgrA LytTR domain. The docking region for savirin was identified as the region between Arg218 on *β*9 and Tyr229 on *β*10 [39]. The report from this study supports the argument that the compounds from this study will aid in preventing biofilm formation. This is because most ligands from this study bind to a similar region of the protein just like savirin; hence, these compounds will display similar inhibition mechanisms as savarin [40]. This can be supported by the reported biofilm inhibition analysis by the study conducted by [23] through in vitro and in vivo studies.

Structural and conformational studies demonstrated that, upon binding by AgrA, the DNA bends to conform to the surface of the protein [41], creating a bend of approximately 38°. This bend is thought to be important for the transcription activation function of AgrA [41]. Protein folding in the AgrA is a result of important salt bridge interactions established among residues in the protein backbone. Nicod et al. [41] reported eight salt bridge interactions needed for protein folding. These salt bridge interactions maintain the structural integrity of the protein to enable protein activation and DNA binding. To understand the mechanism of transcription action by AgrA, elucidating the individual contributions of the residues in the AgrA LytTR domain to transcription activation, a systematic mutational analysis of the AgrA LytTR domain was performed [41]. Findings from this study show that the H174L (His174 mutated to leucine) mutant displayed a reduced ability to activate the P3 promoter by at least 40% compared to WT AgrA since His174 forms salt bridge interactions with Glu226 and Glu163. Interestingly, the essential DNA-binding residue Arg233 was also barely detectable from the scanning, suggesting that this residue is also involved in maintaining the structural fold of the AgrA protein. This is so because Arg233 is directly involved in DNA binding and the protein adapts to a specific structural conformation for binding to occur [41].

Based on the findings, an effective AgrA inhibitor should interact strongly with amino acid residues directly involved in DNA binding, causing perturbation in the distance between His174 and its associated residues crucial for salt bridge formation. Molecular docking results demonstrate that compounds from this study interact strongly with the amino acid residues essential for DNA binding, consistent with previous research. MD simulations further support this, revealing that the ligands maintain strong interactions with the DNA-binding residues. The Rg analysis indicates an increase in the flexibility of the protein when the ligands were bound to the protein as compared to the unbound state (Figure 6(a)). To support these findings, the RMSF (Figure 6(b)) and the PCA (Figure 7) plots demonstrate the major fluctuations of amino acid residues as well as the higher collective motions of atoms in the bound and unbound states. The FEL analysis also supported the results from the Rg, RMSF, and PCA since the energy minima of the protein in the bound states clearly show the instability of the protein [33] conformation throughout the simulation (Figures 8 and 9) since the protein attained two distinct energy minima when the ligands were bound to it.

A computational mutagenesis study identified that mutating residues directly contacting DNA to alanine reduced the protein's affinity for DNA (Figure 10). Trajectory analysis revealed a loss of affinity at ~3.2 ns due to stable distances observed since 0 ns (Figure 10). This loss was attributed to residues indirectly contacting DNA and structural conformation changes. Despite indirect contact, reduced affinity led to detachment. Residues interacting with ligands prevented DNA recognition throughout the simulation (Figure S5). This suggests the potential for inhibitors mimicking these interactions to inhibit biofilm formation by reducing DNA affinity. The results from this study explain the molecular basis of HP antibiofilm activity from an in vitro study conducted by [23].

Considering the importance of the salt bridge interactions between His174-Glu163 and His174-Glu226 for protein folding, the salt bridge interaction analysis was done. From the results, there was instability in the salt bridge interactions in the bound complexes (Figure S6 and Figure 9). These salt bridges stabilize the interactions between the *β*-sheets *β*4 and *β*3 (His174-Glu163) and between the *β*-sheets *β*4 and *β*10 (His174 and Glu226). From a study by [24, 33, 41], these two salt bridge interactions are the only ones taking place on this face of the protein, the only ones linking the *β*-sheet *β*10 to the rest of the protein, and the only ones connecting the *β*-sheets *β*4 and *β*3. Therefore, the destabilization of these salt bridge interactions caused by the ligands when bound to the protein led to a structural conformational change, and this reduced the protein's affinity for DNA. This observation is similar to findings from a study by Nicod et al. when an AgrA His174Leu mutant was barely detected by Western blot using a polyclonal antibody. Again, these findings led to the conclusion that these salt bridge interactions are essential in order for AgrA to adopt the correct structural fold and that residue His174 is thus essential for AgrA function [23].

The results obtained through molecular docking, MD simulations, binding energy calculations, and the computational mutagenesis study show that the mode of action for HP compounds is through their interactions with the AgrA protein at the hydrophobic cleft where DNA-binding amino acids are positioned. Structural conformational analysis demonstrates that upon the ligand binding, the structural integrity of the protein was perturbed, and this indicates the loss of DNA binding and probable biofilm inhibition as observed by Huigens, Abouelhassan, and Yang through their experimental study. Conformational analysis of the protein again highlights the importance of the salt bridge interaction within the protein. One important salt bridge interaction is between His174 with Glu226 and Glu163 since this salt bridge interaction is at the surface of the protein [41]. Future inhibitor design could look into considering molecules that interact with residues responsible for DNA binding. Other studies can also consider exploring other active sites of the protein to examine allostery inhibition of the AgrA protein which would help halt biofilm formation. The computational mutagenesis study also supported important roles played by the residues needed for DNA binding since mutating these residues to alanine resulted in the loss of affinity for the DNA, and this is proven by experimental analysis by [41]. The compounds bind to the loops and specific residues needed for DNA binding and the establishment of interactions that lead to a reduced affinity for the DNA, hence displaying antibiofilm activity in *S. aureus*, as also demonstrated by Huigens, Abouelhassan, and Yang through an in vitro experiment.

The binding free energy analysis for the AgrA-DNA complex with various compounds (C1–C7, HP, and HP-14) reveals key insights into the molecular interactions influencing binding affinities. All compounds enhance the binding affinity compared to the apo form, which has a Δ*G*_bind_ of −80.75 kcal/mol. The strongest binding affinities are observed with C7 (−113.84 kcal/mol), HP-14 (−115.23 kcal/mol), and HP (−112.28 kcal/mol). These results underscore the importance of hydrophobic and electrostatic interactions in designing effective inhibitors for the AgrA-DNA complex. Compounds like C7, HP-14, and HP serve as promising lead compounds for further optimization. Overall, this analysis provides a foundation for rational drug design targeting the AgrA-DNA interaction, with potential implications for developing new treatments.

## 5. Conclusion

This study explored the antibiofilm potential of HP compounds against *S. aureus* by targeting the AgrA protein, a key regulator of biofilm formation. Through molecular docking, MD simulations, and computational mutagenesis, it was found that these compounds bind to the AgrA protein at the hydrophobic cleft where critical DNA-binding amino acids are located. This binding disrupted essential salt bridge interactions between His174-Glu163 and His174-Glu226, leading to conformational changes that reduced the protein's affinity for DNA. The findings are consistent with previous experimental studies that highlight the importance of these residues and salt bridge interactions in maintaining AgrA's structural integrity and function. Binding free energy analysis for the AgrA-DNA complex with various compounds (C1–C7, HP, and HP-14) revealed key insights into the molecular interactions influencing binding affinities. All compounds enhanced the binding affinity compared to the apo form, which has a Δ*G*_bind_ of −80.75 kcal/mol. The strongest binding affinities were observed with C7 (−113.84 kcal/mol), HP-14 (−115.23 kcal/mol), and HP (−112.28 kcal/mol). These results underscore the importance of hydrophobic and electrostatic interactions in designing effective inhibitors for the AgrA-DNA complex. Compounds like C7, HP-14, and HP serve as promising lead compounds for further optimization. The compounds' ability to perturb the structural conformation of AgrA suggests a promising mechanism for inhibiting biofilm formation by preventing DNA binding. This mechanism mirrors the action of savirin, a known AgrA inhibitor, and supports the potential of HP compounds as effective antibiofilm agents. The results obtained from this study provide a molecular basis for the antibiofilm activity observed in vitro by Huigens, Abouelhassan, and Yang and pave the way for further exploration of these compounds as novel therapeutic agents against *S. aureus* biofilms. Future research should focus on designing inhibitors that target key residues involved in DNA binding and exploring other active sites on the protein to uncover allosteric inhibition mechanisms. These findings contribute significantly to understanding the molecular interactions crucial for AgrA function and offer valuable insights for developing new strategies to combat biofilm-associated infections. Overall, this analysis provides a foundation for rational drug design targeting the AgrA-DNA interaction, with potential implications for developing new treatments.

## Figures and Tables

**Table 1:** Binding affinities of compounds considered for this study.

**Compounds**	**Binding affinity (kcal/mol)**
C1	−9.8
C2	−9.7
C3	−9.8
C4	−9.8
C5	−9.4
C6	−8.0
C7	−10.0
C8	−8.9
HP-14	−10.0
HP	−9.8

**Table 2:** MM-PBSA calculations of binding energy for apo _(AgrA-DNA)_ and (AgrA-DNA)+Ci. Ci represents compounds named from C1 to C7 and HP represents halogenated phenazine.

**Complex**	Δ**E**_**vdW**_	Δ**E**_**elect**_	Δ**G**_**PB**_	Δ**G**_**SASA**_	Δ**G**_**bind**_
Apo _(AgrA-DNA)_	−68.73	−38.68	48.76	−22.10	−80.75
(AgrA-DNA)+C1	−115.82	−24.33	63.95	−32.04	−104.36
(AgrA-DNA)+C2	−112.53	−25.68	65.86	−31.42	−103.77
(AgrA-DNA)+C3	−120.68	−23.10	67.35	−30.23	−106.66
(AgrA-DNA)+C4	−122.90	−23.76	69.80	−30.50	−107.36
(AgrA-DNA)+C5	−120.43	−25.04	70.68	−30.97	−105.76
(AgrA-DNA)+C6	−126.19	−25.00	71.24	−30.63	−110.58
(AgrA-DNA)+C7	−135.81	−28.92	81.56	−30.67	−113.84
(AgrA-DNA)+HP	−130.75	−28.56	79.34	−32.31	−112.28
(AgrA-DNA)+HP-14	−131.39	−26.87	73.26	−30.23	−115.23

## Data Availability

All data generated or analyzed during this study are included in this published article.

## Supporting Information

Supporting Information Additional supporting information can be found online in the Supporting Information section. Figure S1: Compound C4 also binding in a similar region just like the other compounds. Figure S2: C5 demonstrated a different binding site after the virtual screening. Figure S3: RMSD for protein backbone during the simulation period. Figure S4: FEL for other compounds considered for the study. Figure S5: distance between AgrA LytTR and DNA for the ligand-bound complexes, mutants, and apoproteins (wild type). Figure S6: distance Glu163-His174 salt bridge interaction throughout the simulation. Figure S7: the newly discovered residue (Lys187) which helps in DNA binding.
