# Supplementary material for: Computational Mutagenesis and Inhibition of Staphylococcus aureus AgrA LytTR Domain Using Phenazine Scaffolds: Insight From a Biophysical Study
Source: Biomed Res Int. 2024 Sep 18;2024:8843954. doi: 10.1155/2024/8843954 (PMC11424843; doi:10.1155/2024/8843954)
Supplement: Supporting Information — Additional supporting information can be found online in the Supporting Information section. Figure S1: Compound C4 also binding in a similar region just like the other compounds. Figure S2: C5 demonstrated a different binding site after the virtual screening. Figure S3: RMSD for protein backbone during the simulation period. Figure S4: FEL for other compounds considered for the study. Figure S5: distance between AgrA LytTR and DNA for the ligand-bound complexes, mutants, and apoproteins (wild type). Figure S6: distance Glu163-His174 salt bridge interaction throughout the simulation. Figure S7: the newly discovered residue (Lys187) which helps in DNA binding. [file 8843954.f1.docx]

**Supplementary Information**


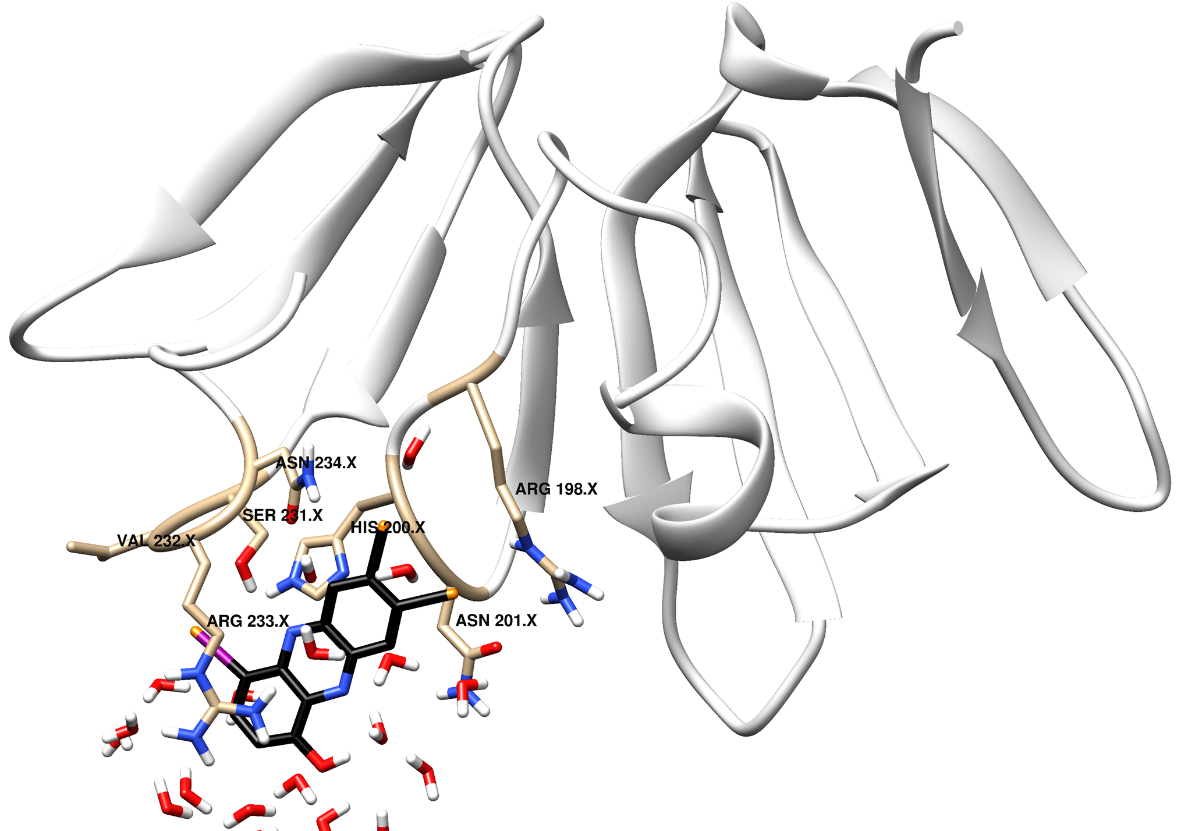


**Figure S1**: Compound C4 also binding at the similar region just like the other compounds.


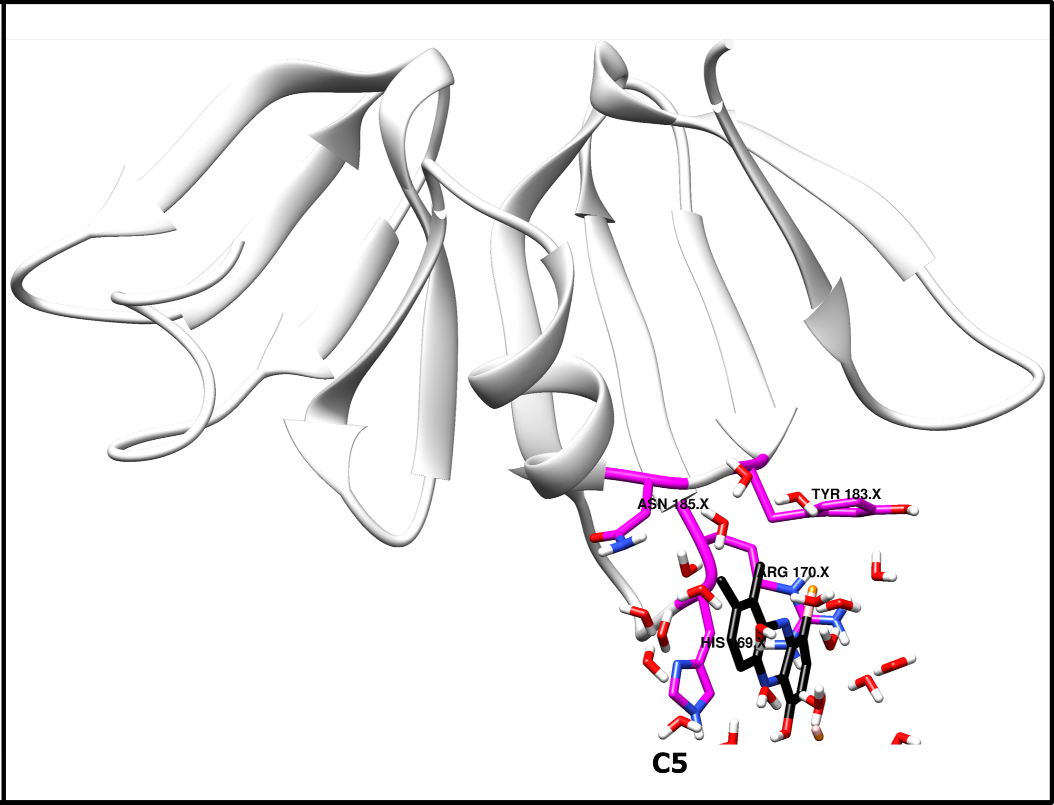


**Figure S2**: C5 demonstrated a different binding site after virtual screening.


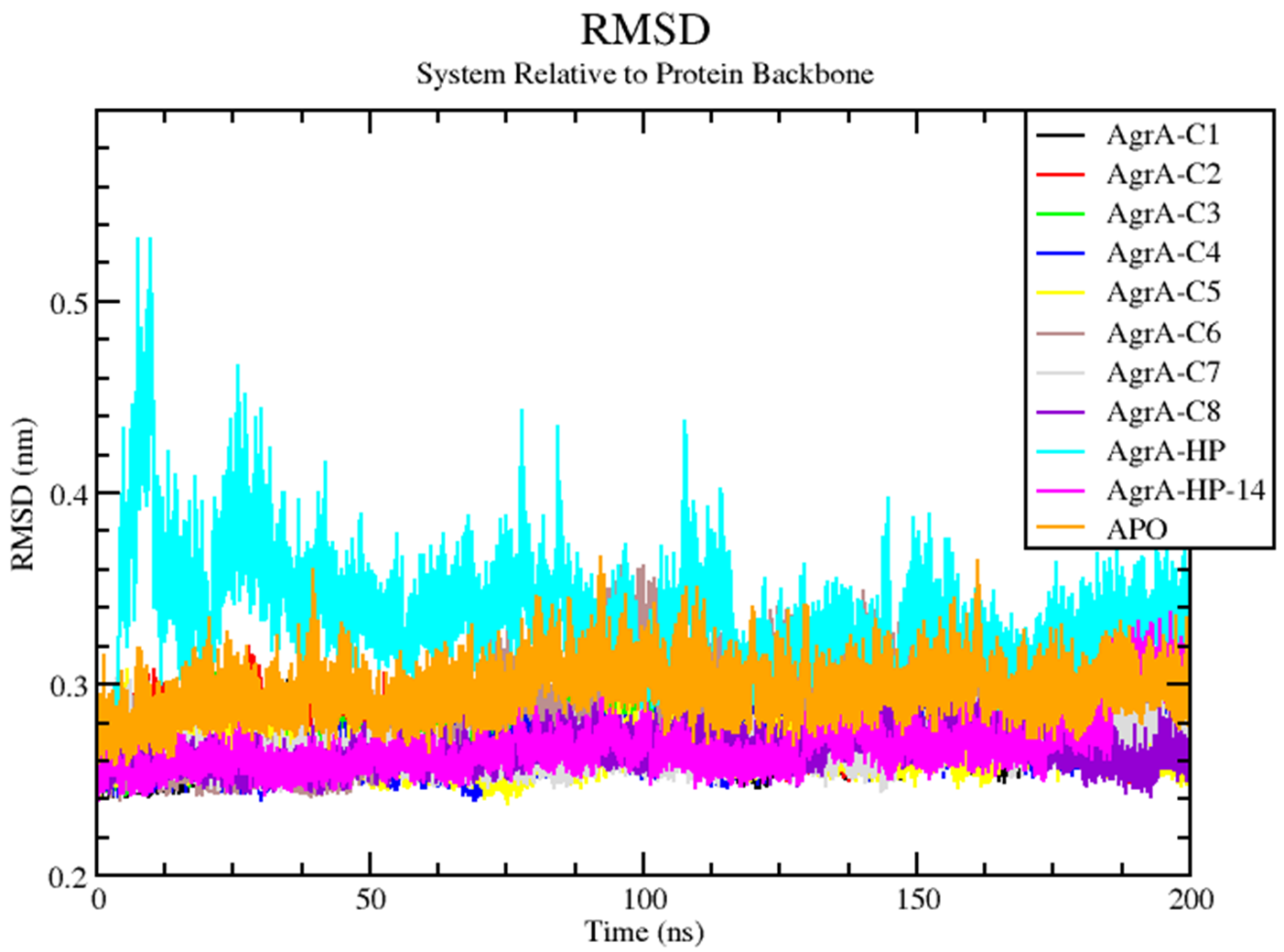


**Figure S3**: RMSD for protein backbone during the simulation period.


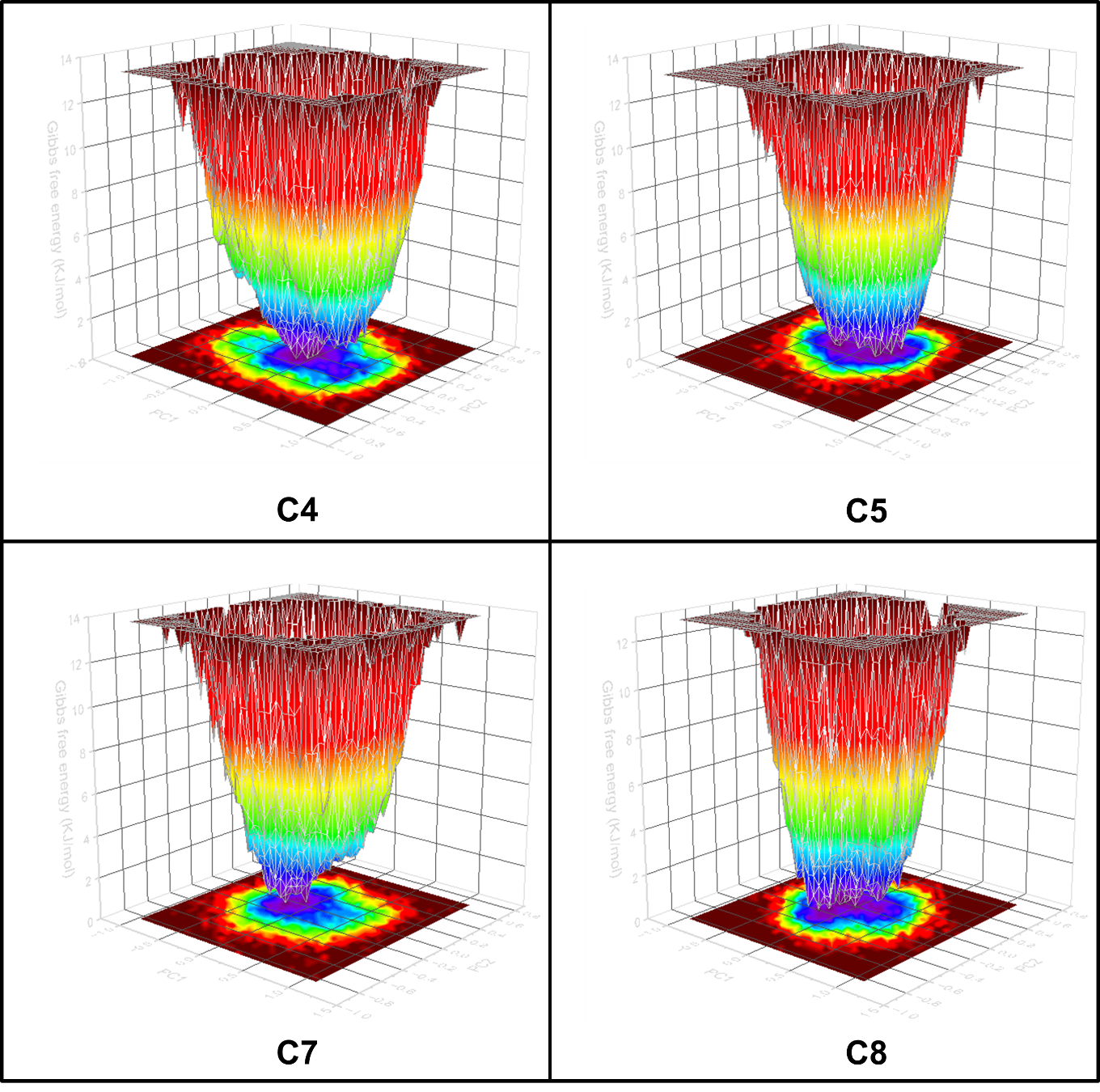


**Figure S4**: FEL for other compounds considered for the study.


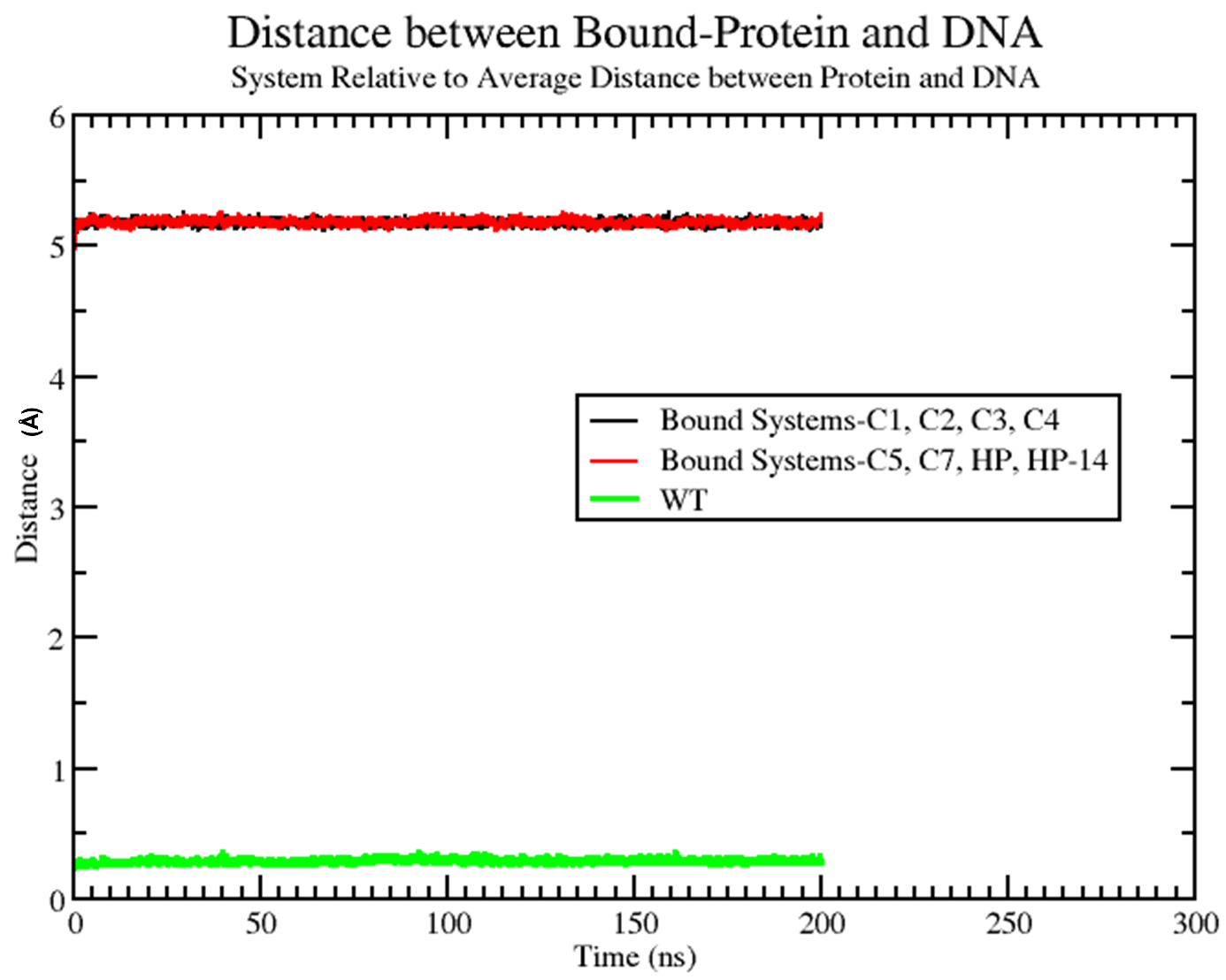


**Figure S5**: Distance between AgrA LytTR and DNA for the ligand bound complexes, mutants and apoprotein (wild-type).


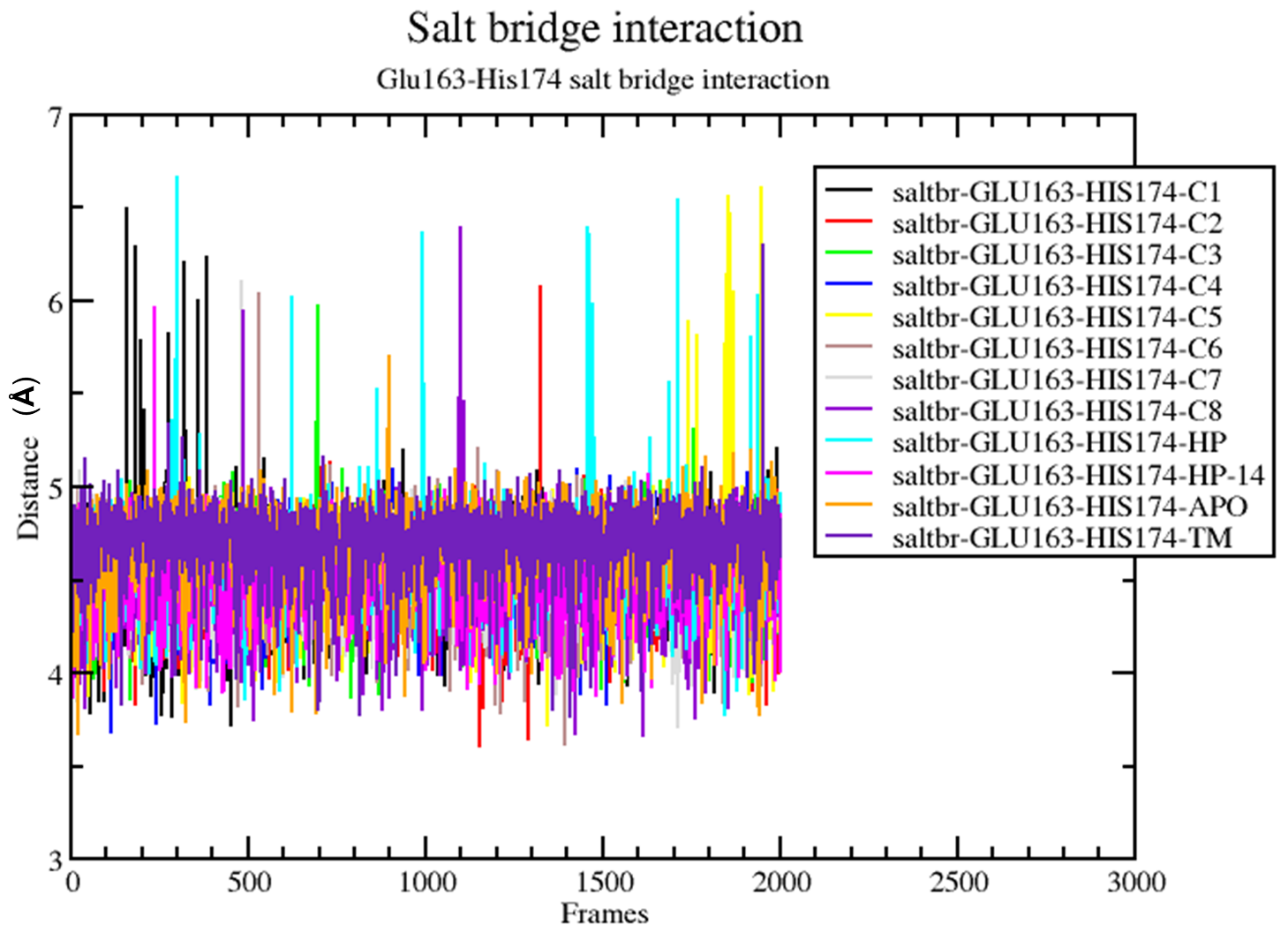


**Figure S6**: Distance Glu163-His174 salt-bridge interaction throughout the simulation.


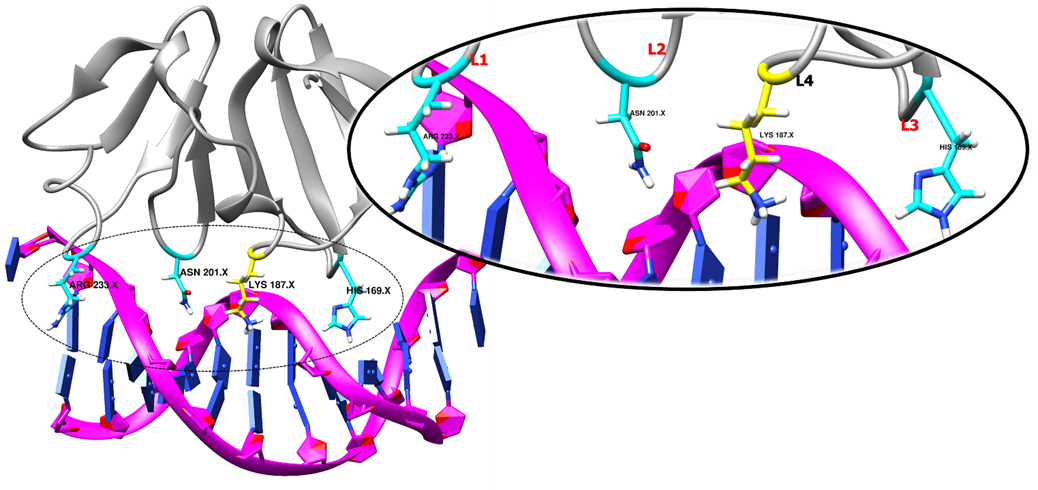


**Figure S7**: The new discovered residue (Lys187) which helps in DNA binding.
